# Effects of host vimentin on *Eimeria tenella* sporozoite invasion

**DOI:** 10.1186/s13071-021-05107-4

**Published:** 2022-01-04

**Authors:** Zhan Liu, Xiangfei Geng, Qiping Zhao, Shunhai Zhu, Hongyu Han, Yu Yu, Wenhao Huang, Yawen Yao, Bing Huang, Hui Dong

**Affiliations:** 1 Key Laboratory of Animal Parasitology of Ministry of Agriculture, Shanghai Veterinary Research Institute, Chinese Academy of Agricultural Sciences, Minhang, 200241 Shanghai People’s Republic of China; 2 Beijing YuanDa Spark Medicine Technology Co., Ltd, Beijing, 100088 People’s Republic of China

**Keywords:** *Eimeria*, Invasion, Vimentin, Invasion assay

## Abstract

**Background:**

Chicken coccidiosis is a parasitic disease caused by *Eimeria* of Apicomplexa, which has caused great economic loss to the poultry breeding industry. Host vimentin is a key protein in the process of infection of many pathogens. In an earlier phosphorylation proteomics study, we found that the phosphorylation level of host vimentin was significantly regulated after *Eimeria tenella* sporozoite infection. Therefore, we explored the role of host vimentin in the invasion of host cells by sporozoites.

**Methods:**

Chicken vimentin protein was cloned and expressed. We used qPCR, western blotting, and indirect immunofluorescence to detect levels of mRNA transcription, translation, and phosphorylation, and changes in the distribution of vimentin after *E. tenella* sporozoite infection. The sporozoite invasion rate in DF-1 cells treated with vimentin polyclonal antibody or with small interfering RNA (siRNA), which downregulated vimentin expression, was assessed by an in vitro invasion test.

**Results:**

The results showed that vimentin transcription and translation levels increased continually at 6–72 h after *E. tenella* sporozoite infection, and the total phosphorylation levels of vimentin also changed. About 24 h after sporozoite infection, vimentin accumulated around sporozoites in DF-1 cells. Treating DF-1 cells with vimentin polyclonal antibody or downregulating vimentin expression by siRNA significantly improved the invasion efficiency of sporozoites.

**Conclusion:**

In this study, we showed that vimentin played an inhibitory role during the invasion of sporozoites. These data provided a foundation for clarifying the relationship between *Eimeria* and the host.

**Graphical Abstract:**

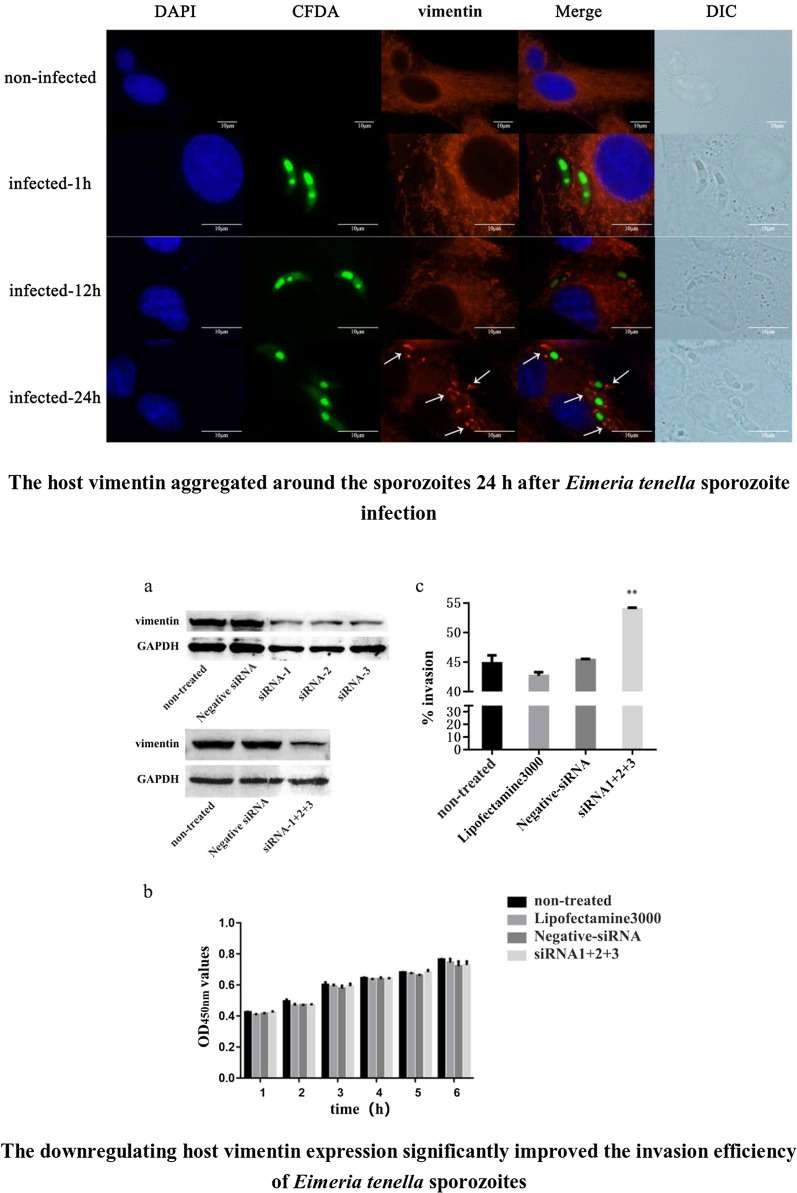

**Supplementary Information:**

The online version contains supplementary material available at 10.1186/s13071-021-05107-4.

## Background

Coccidiosis in chickens is caused by infection with parasites of the genus *Eimeria* and is one of the most common and damaging parasitic diseases in poultry farming, causing economic losses of approximately £2 billion a year worldwide [1]. Conventional methods for the prevention and control of coccidiosis include anti-coccidian drugs and live vaccines; however, these measures give rise to several issues with regard to drug resistance, food security, production cost, and the cross-protection of species [2–4]. Therefore, there is an urgent need to seek new ideas and perspectives on the prevention and control of coccidiosis.

*Eimeria tenella* has the strongest pathogenicity among the known chicken *Eimeria* species. As an obligate intracellular parasite, *E. tenella* must rely on host cells to complete the whole life cycle [5]. Therefore, the identification of key proteins involved in the invasion process is particularly important for exploring the molecular mechanism of coccidian infection and finding a breakthrough method to prevent and control coccidiosis [6].

The mechanism of *E. tenella* invasion in host cells has been explored for many years. Research developments have provided growing evidence that the *E. tenella* infection process is not the independent behavior of the parasite, but a complex process with the participation of host cells. For instance, invasion of host cells by *E. tenella* causes host F-actin-specific aggregation on the surface of sporozoites. This aggregation is closely related to the invasion efficiency of *E. tenella*, and the inhibition of F-actin aggregation by cytochalasin D will reduce the invasion rate of *E. tenella* [7]. *Eimeria tenella* sporozoites can be attached to the intestinal epithelium through the duodenal mucin of the chicken, and host proteins can significantly inhibit the invasion of the cells by sporozoites [8]. The expression of host fatty acid-binding protein 4 increased significantly after 72 h of invasion by *E. tenella* sporozoites, and the overexpression of this protein significantly inhibited the invasion [9]. Processing of the host cells with polyclonal antibodies against the receptor for activated C kinase 1 can promote the invasion of *E. tenella* sporozoites [10]**.** These results demonstrate that host proteins play an indispensable role in *E. tenella* invasion.

In our previous study, we used tandem mass tag-labeled quantitative phosphorylation proteomic technology and parallel reaction monitoring to screen and verify the significantly regulated phosphoproteins of chicken embryo fibroblast cell line (DF-1) cells infected with *E. tenella* sporozoites. The results showed that the phosphorylation level of host vimentin changed significantly after infection. The phosphorylation levels of vimentin at Ser6 and Ser7 sites were significantly higher than those of the uninfected cells 6 h and 36 h after infection by sporozoites, while the phosphorylation level at Ser 4 sites was significantly lower 36 h after infection than after 6 h (unpublished data). Host vimentin is a key protein in the process of infection of many pathogens, but its role differs among different pathogens [11–14], and its role in *Eimeria* infection has not been explored. Therefore, we explored the role of host vimentin in the invasion of host cells by *E. tenella* sporozoites.

## Methods

### Parasites, cells, and experimental animals

The Shanghai strain of *E. tenella* (CAAS21111611) used in the current study has been retained in our laboratory since 1993 and was propagated by passage through 14-day-old coccidia-free chickens [15]. Sporozoites were collected and purified from cleaned sporulated oocysts using standard procedures [16, 17]. DF-1 cells (ATCC CRL-12203) were cultured in Dulbecco’s modified Eagle’s medium (DMEM) (Gibco BRL, Paisley, UK) supplemented with 10% fetal bovine serum (FBS) (Gibco) and 1% penicillin/streptomycin (PS) (Gibco) at 37 °C in a 5% CO_2_ incubator. Chinese yellow chickens were purchased from Shanghai Fuji Biotechnology Co., Ltd., and kept in an environment without coccidia.

### Cloning and identification of the chicken vimentin gene

The total RNA in DF-1 cells was extracted using a MiniBEST Universal RNA Extraction Kit (TaKaRa, Dalian, China) and the first-strand cDNA was prepared using total RNA and a reverse transcription kit (TaKaRa). The primers were designed according to the coding region (1383-base-pair [bp]) sequence of the *Gallus gallus* (chicken) vimentin gene (GenBank accession number: NM_001048076.2) as follows: forward primer: 5′-CGGAATTCATGAGCTTCACCAGCAGCAA-3′, reverse primer: 5′-CCCTCGAGTTACTCCAAGTCATCGTGATGCTG-3ʹ, including *EcoRI* and *XhoI* restriction enzyme cutting site, respectively (underlined label). The chicken vimentin gene was amplified and cloned into the expression vector pGEX-4 T-1 to construct the recombinant plasmid pGEX-4 T-vimentin. Then the recombinant plasmid was transformed into *Escherichia coli* TOP10 competent cells (Tiangen Biotech, Beijing, China) and selected with Luria-Bertani (LB) solid medium containing ampicillin. The positive-screened colonies were sent to the Shanghai Qingke Company for sequencing. Basic Local Alignment Search Tool (BLAST) programs at the National Center for Biotechnology Information (http://www.ncbi.nlm.nih.gov/BLAST/) were used for the similarity analysis of the sequencing results and vimentin gene full-length sequences.

### Expression, purification, and identification of recombinant vimentin protein

The verified recombinant plasmid was transformed into *E. coli* BL21(DE3) competent cells (Tiangen) and cultured in a shaker of 37 °C and 180 rpm. When the OD_600_ reached about 0.6, IPTG was added (final concentration 1 mmol/l; Sigma-Aldrich, St. Louis, MO, USA) to induce and express recombinant vimentin protein. The cell pellet was collected by centrifugation and lysed with sonication at 4 °C. Then the lysed product was analyzed by 10% sodium dodecyl sulfate–polyacrylamide gel electrophoresis (SDS-PAGE) to confirm that the recombinant protein was present as a soluble protein or in inclusion bodies. The recombinant protein was purified according to the characteristics using a published gel purification method [18]. Purified recombinant vimentin protein (10 μg) was analyzed by SDS-PAGE and transferred to polyvinylidene fluoride (PVDF) membrane (Millipore, Billerica, MA, USA). The PVDF membrane was blocked in 5% skim milk for 2 h at 37 °C and incubated with Anti-GST Tag Mouse Monoclonal Antibody (1:2000; CWBio, Beijing, China) for 2 h at 37 °C, and was then washed in phosphate-buffered saline (PBS) three times for 5 min and incubated with peroxidase-conjugated AffiniPure Goat Anti-Mouse IgG (H+L) (1:5000; Proteintech, Chicago, IL, USA) for 45 min at room temperature. Finally, imaging was obtained using the ChemiDoc Touch Imaging System (Bio-Rad, Hercules, CA, USA).

### Collection of DF-1 cells infected or not infected with *E. tenella* sporozoites

DF-1 cells (1.5 × 10^6^ cells/well) were plated into six-well plates and cultured at 37 °C in a 5% CO_2_ incubator to a cell coverage of 80–90%. Freshly isolated sporozoites were incubated with DMEM (2% FBS, 5% PBS) for 2 h at 37 °C. The DF-1 cells were infected with pretreated sporozoites with a multiplicity of infection of 3 (MOI = 3). At different time points during infection (1, 2, 6, 12, 24, 36, 48, 60, and 72 h), the medium and noninvasive sporozoites were removed by washing gently with PBS three times. The DF-1 cells without infecting sporozoites were set as the uninfected control. Every cell sample was harvested with a cell scraper and stored at −80 °C for subsequent analysis.

### Assessment of vimentin mRNA transcription levels after sporozoite infection

Total RNA was extracted from cells collected from different time points using the MiniBEST Universal RNA Extraction Kit (TaKaRa) according to the manufacturer’s instructions. The design of specific primers was based on published vimentin and GAPDH gene sequences, as follows: vimentin, forward primer: 5′-GCAAAGTTGAGTCCCTGCAA-3′, reverse primer: 5′-AGGGCAGCAGTAAGATCAGG-3′; GAPDH, forward primer: 5′-GGCACTGTCAAGGCTGAGAACG-3′, reverse primer: 5′- TGAGATGATAACACGCTTAGCACCAC-3′. Vimentin mRNA transcription levels in each group of samples were determined using the One-Step TB Green PrimeScript RT-PCR Kit II (TaKaRa), according to the manufacturer’s instructions. Each reaction was carried out in triplicate, and the experiment was performed three times. The relative expression of vimentin mRNA was calculated using the 2^−ΔΔCt^ method [19], and SPSS 22.0 (https://www.ibm.com) was used for t-test analysis.

### Analysis of vimentin expression levels after sporozoite infection

Western blotting was used to detect vimentin expression levels during sporozoite infection. Proteins at different time points (6, 36, and 72 h) were extracted from infected and uninfected samples using cell lysis buffer IP (Beyotime, Haimen, China) for western blotting. Protein concentration was determined by a BCA Protein Assay Kit (Beyotime), then 10 μg of protein was taken from each group for SDS-PAGE and transferred to PVDF membrane. The PVDF membrane was blocked in 5% skim milk for 2 h at 37 °C and then incubated with polyclonal rabbit antibody against vimentin (1:200, prepared by our laboratory) or β-GAPDH (internal reference, 1:2000; Yeasen, Shanghai, China) for 2 h at 37 °C. They were then washed in PBS three times for 5 min and incubated with goat anti-rabbit IgG antibody (1:5000; LI-COR Biosciences, Lincoln, NE, USA) for 45 min at room temperature. Finally, images were obtained with the ChemiDoc Touch Imaging System.

### Determination of vimentin phosphorylation levels after sporozoite infection

Proteins were extracted from infected and uninfected cells collected from different time points (1, 2, 6, 12, 24, 36, 48, 60, and 72 h) using a phosphorylated protein extraction kit (Solarbio, Beijing, China). Protein concentration was determined using a BCA protein assay kit (Beyotime), and 10 μg of protein from each group was used for Phos-tag acrylamide-based gel (Wako, Tokyo, Japan) analysis. After electrophoresis, Phos-tag acrylamide gels were immersed three times in transfer buffer containing 1 mM EDTA for 10 min and then washed in transfer buffer without EDTA for 10 min. Then the proteins were transferred to a PVDF membrane for western blot analysis.

### Assessment of vimentin distribution after sporozoite infection

Cell climbing films were placed in six-well plates. DF-1 cells (1.5 × 10^6^ cells/well) were plated into the six-well plates and cultured to a cell coverage of 80%–90%. Sporozoites were labeled with PBS containing carboxyfluorescein diacetate succinimidyl ester (CFSE) (1:20,000; Invitrogen, Carlsbad, CA, USA) for 15 min and then incubated with DMEM (2% FBS, 5% PS) for 2 h at 37 °C. Labeled sporozoites were added to the DF-1 cells with a multiplicity of infection of 3 (MOI = 3). Samples were collected from the six-well culture plates at different time points (1, 12, and 24 h). The uninfected and infected DF-1 cells were fixed with 4% paraformaldehyde (Solarbio) for 15 min, permeabilized with 0.1% Triton X-100 for 15 min, and blocked in 2% bovine serum albumin (BSA) (Solarbio) at 4 °C for 12 h. DF-1 cells were incubated with polyclonal rabbit antibody against vimentin (1:200) for 2 h at 37 °C, and then washed in PBS three times for 5 min and incubated with Alexa Fluor 647 Goat Anti-Rabbit IgG (1:500; Invitrogen) for 45 min at room temperature. DF-1 cells were washed again with PBS, stained with DAPI (1:500; Beyotime) for 15 min, treated with Fluoromount Aqueous Mounting Medium (Sigma-Aldrich), and imaged under a fluorescence microscope (Olympus, Tokyo, Japan).

### Analysis of anti-vimentin polyclonal antibodies on sporozoite invasion

DF-1 cells (3.0 × 10^5^ cells/well) were plated into 24-well plates and cultured to a cell coverage of 80–90%. Polyclonal rabbit antibody against vimentin (prepared by our laboratory) was purified using protein A+G agarose (Beyotime). Then, purified IgG was added to DF-1 cells at a final concentration of 25, 50, 100, 200, 300, or 400 μg/ml in DMEM (2% FBS, 5% PS) at 37 °C for 3 h, respectively. The same quantity of purified IgG from rabbit sera was used as negative control, and DF-1 cells incubated without antibodies were used as a positive control. After 3 h, the original medium was replaced with DMEM (2% FBS, 5% PS), and the sporozoite invasion rate was determined by an invasion assay in vitro [9]*.* Labeled sporozoites (as described above) were added to DF-1 cells at a multiplicity of infection of 3 (MOI = 3). After invasion for 6 h at 37 °C in 5% CO_2_, DF-1 cells were washed with PBS to remove noninvasive sporozoites, digested with trypsin, and analyzed by flow cytometry (Cytomics FC 500; Beckman Coulter, USA). A total of three tests were carried out.

### Effects of downregulation of vimentin expression by small interfering RNA (siRNA) on sporozoite invasion ability

Transfection experiments were performed when cell coverage reached 40–50% using the siRNA sequences published by Schäfer et al. (Table 1) [20]. The DF-1 cells were transfected with siRNA 1 + 2 + 3 or negative siRNA using Lipofectamine 3000 (Invitrogen) according to the manufacturer’s instructions. Briefly, DNA and the transfection reagent were mixed (10 ml lipofectamine 3000 and 4 μg DNA), incubated at room temperature for 30 min, and added to the cells. Six hours later, the DNA-transfection reagent mixture was replaced by DMEM containing 10% FBS. All plasmids were transfected in triplicates. In the mock-treated cells, only the transfection reagent was used. At 24 h post-transfection, the protein samples were collected to test vimentin expression levels by western blot analysis.

**Table 1:** Sequences of siRNA used to downregulate the vimentin expression

siRNA no.	siRNA sequences
siRNA-1	5′-CCAUCAACACGGAGUUCAATT-3′
siRNA-2	5′-CCGACAGGAUGUUGACAAUTT-3′
siRNA-3	5′-GGAAGAAAUGGCUCGCCAUTT-3′
Negative siRNA	5′-UUCUCCGAACGUGUCACGUTT-3′

In order to determine the cell growth after transfection with siRNAs, we examined cell activity. DF-1 cells (0.8 × 10^5^ cells) were plated into 96-well plates, then transfected using the above method. The original medium was discarded after 24 h transfection, 90 μl complete medium and 10 μl CCK-8 solution were added to each well, and then the OD_450_ value of the cells was measured every hour for 6 h.

DF-1 cells (3.0 × 10^5^ cells) were plated into 24-well plates and transfected with siRNA. After 24 h transfection, the CFSE-labeled sporozoites (as described above) were added to DF-1 cells at a multiplicity of infection of 3 (MOI = 3). After invasion for 6 h at 37 °C in 5% CO2, DF-1 cells were washed with PBS to remove noninvasive sporozoites, digested with trypsin, and analyzed by flow cytometry (Cytomics FC 500; Beckman Coulter, USA). DF-1 cells that were not transfected and transfected with negative siRNA were used as control groups. A total of three tests were carried out.

### Statistical analysis

The experimental data were compared using SPSS version 22.0 statistical software (SPSS IBM Corp., Armonk, NY, USA). The *t*-test was used for two independent samples, and one-way analysis of variance (ANOVA) and Duncan’s multiple range test were used for multiple groups. Significance was set at *P* < 0.05, with *P* < 0.01 indicating an extremely significant difference. GraphPad Prism 5 (GraphPad Software Inc., San Diego, CA, USA) was used for graphing the results.

## Results

### Expression, purification, and validation of recombinant vimentin protein

A band of about 1383 bp was obtained by polymerase chain reaction (PCR) amplification. A BLASTN comparison indicated that the obtained sequence was 99.9% homologous with the published *G. gallus* vimentin gene (GenBank accession number: NM_001048076.2). The vimentin gene encoded a 460 aa protein with a molecular mass of about 53.2 kDa. A protein band of about 80 kDa was observed in SDS-PAGE because a 26 kDa GST tag was derived from the vector. The recombinant vimentin protein was purified by gel purification and recognized by anti-GST tag mouse monoclonal antibody (Additional file 1: Figure S1).

### Vimentin mRNA transcription levels after *E. tenella* sporozoite infection

**Fig. 1:**
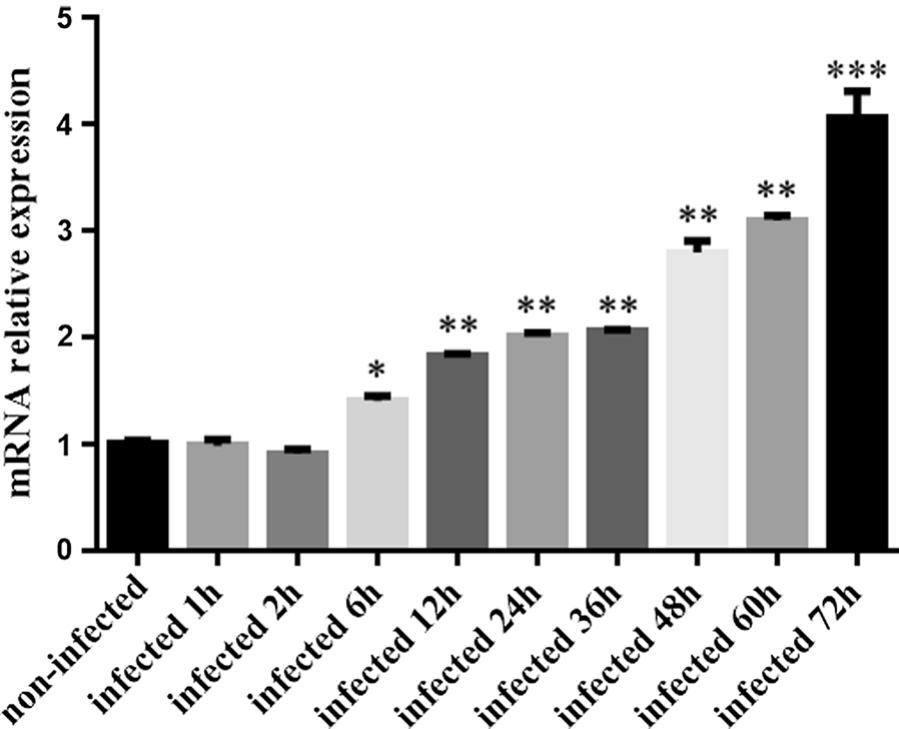
Vimentin mRNA transcription levels in *E. tenella* sporozoite-infected DF-1 cells. *P < 0.05, ** < 0.01 and *** < 0.001 by Student’s *t-*test compared with the uninfected group

The mRNA levels of vimentin in DF-1 cells infected for 1, 2, 6, 12, 24, 36, 48, 60, and 72 h and uninfected *E. tenella* sporozoites were measured using real-time quantity PCR (qPCR). The results showed that the transcriptional level of vimentin increased significantly 6–72 h after sporozoite infection (Fig. 1) (*F*_(7,16)_ = 244.681, *P* < 0.0001). As compared with the uninfected group, the transcriptional levels of vimentin in the 1 h and 2 h infected groups were not significant (*F*_(2,6)_ = 2.625, *P* = 0.152), that of in the 6 h infected group was higher (*F*_(1,4)_ = 13.500, *P* = 0.021), and those of in 12–72 h infected groups increased significantly (*F*_(6,14)_ = 241.864, *P* < 0.0001).

### Vimentin expression levels after *E. tenella* sporozoite infection

**Fig. 2:**
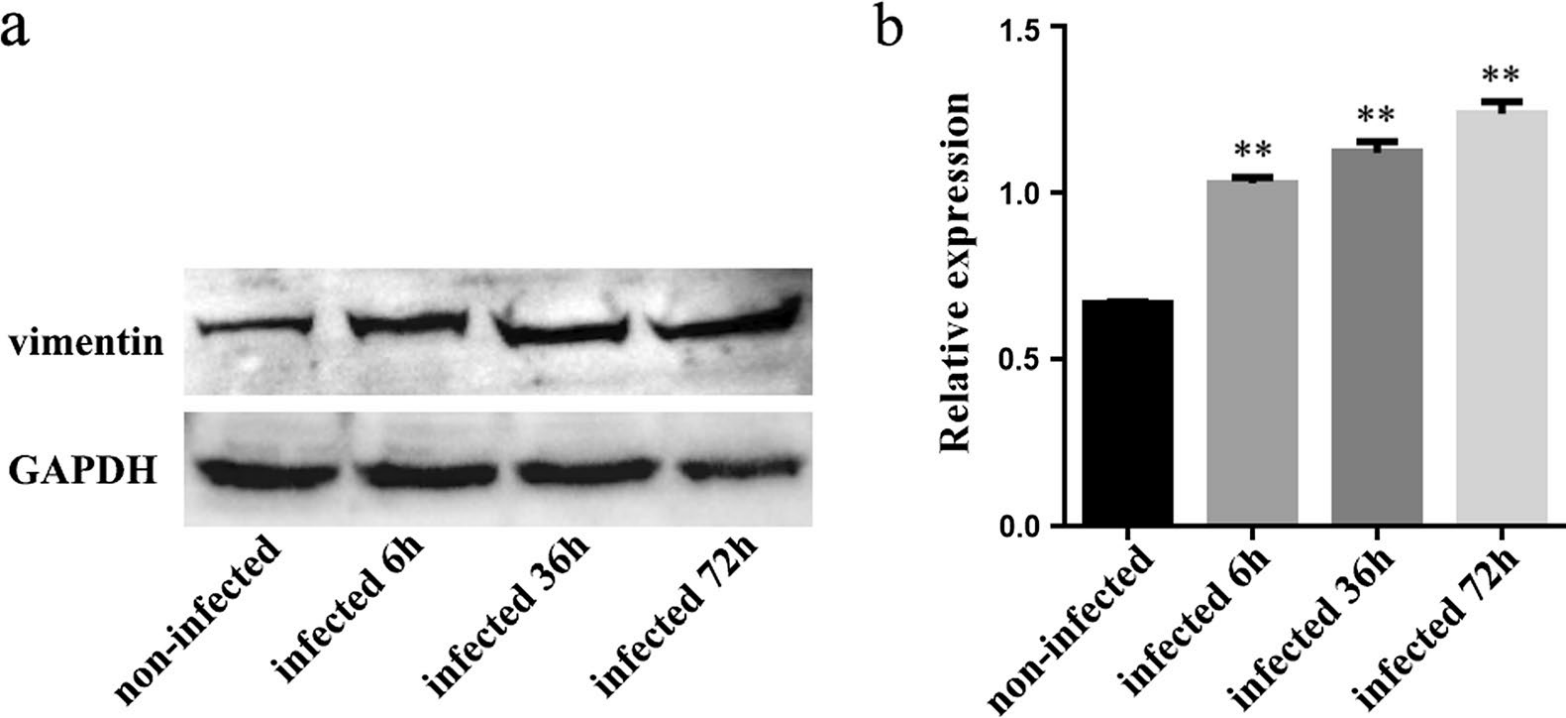
Vimentin protein expression levels in *E. tenella* sporozoite-infected DF-1 cells. **a** Western blotting of the internal reference GAPDH and vimentin protein. **b** Gray analysis of the relative expression level of the vimentin protein. **P < 0.01 by Student’s *t*-test compared with the uninfected group

The protein expression levels of vimentin in DF-1 cells infected for 6, 36, and 72 h and uninfected *E. tenella* sporozoites were measured using western blotting. The results showed that the protein expression levels of vimentin increased significantly 6–72 h after sporozoite infection (Fig. 2) (*F*_(3,8)_ = 20.204, *P* < 0.0001).

### Total phosphorylation levels of vimentin after *E. tenella* sporozoite infection

**Fig. 3:**
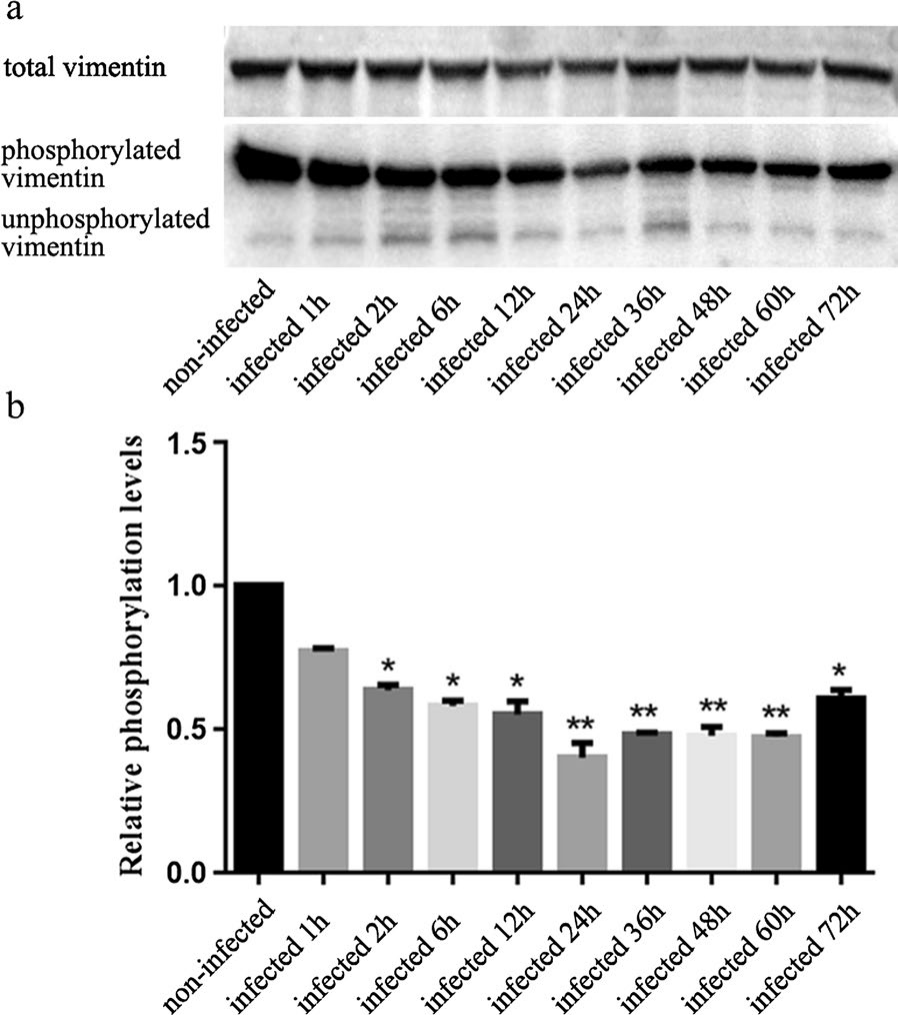
Total phosphorylation levels of vimentin in *E. tenella* sporozoite-infected DF-1 cells. **a** Western blotting of phosphorylated and unphosphorylated vimentin with a consistent level of total vimentin. **b** Gray analysis of the relative expression level of phosphorylated vimentin. *P < 0.05 and **P < 0.01 Student’s *t*-test compared with the uninfected group

Total phosphorylation levels of vimentin in DF-1 cells infected for 1, 2, 6, 12, 24, 36, 48, 60, and 72 h and uninfected *E. tenella* sporozoites were measured using western blotting. The results showed that the total phosphorylation levels of vimentin in DF-1 cells decreased within 24 h of infection and reached the lowest level at 24 h of infection, and then slightly increased (Fig. 3) (*F*_(9,20)_ = 16.362, *P* < 0.0001).

### Vimentin distribution in DF-1 cells infected by *E. tenella* sporozoites

**Fig. 4:**
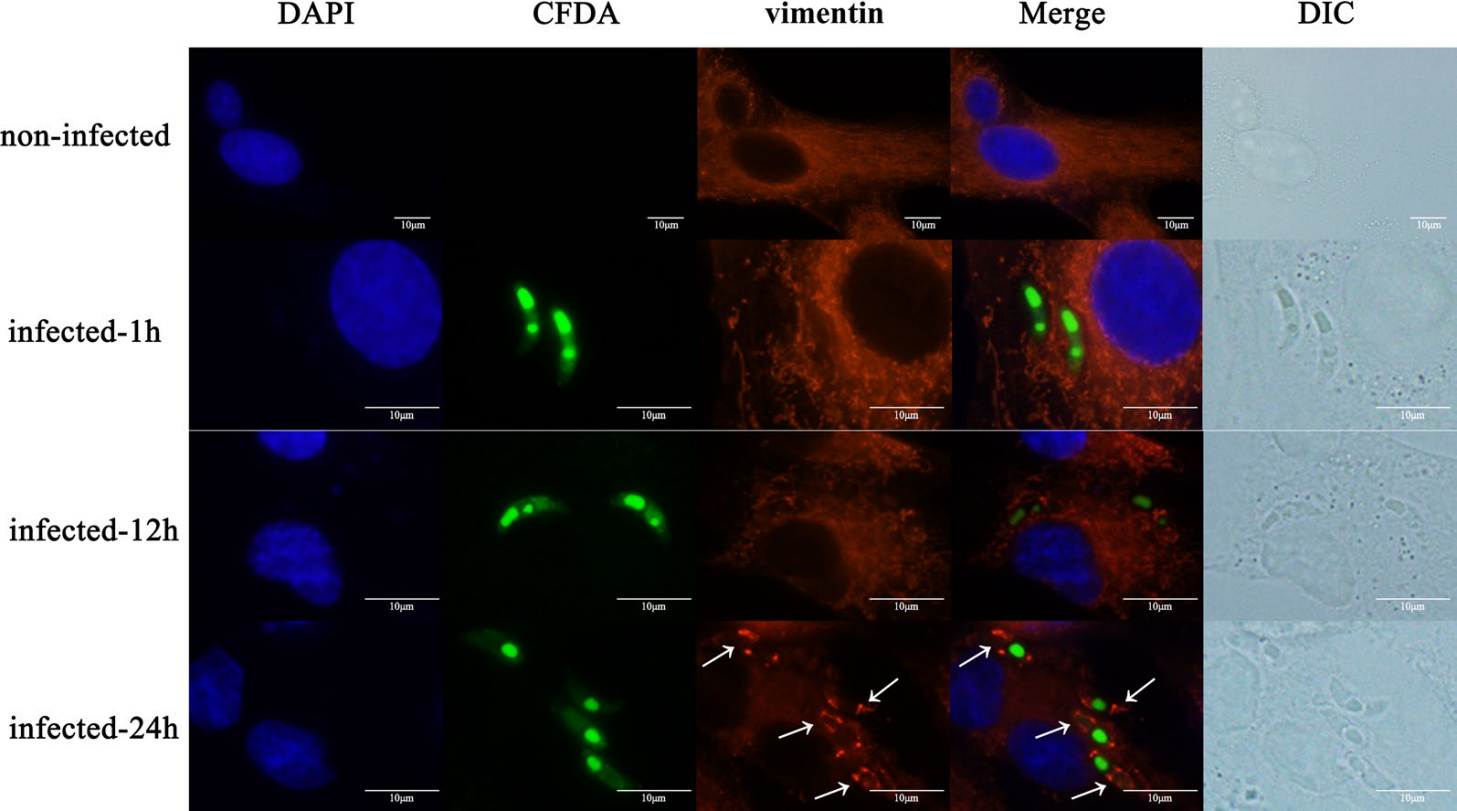
Changes in vimentin distribution after *E. tenella* sporozoite invasion in DF-1 cells

The distribution of vimentin in DF-1 cells infected with *E. tenella* sporozoites for 1, 12, and 24 h and in uninfected DF-1 cells was determined using indirect immunofluorescence. The results showed that vimentin aggregated around the sporozoites 24 h after sporozoite infection (Fig. 4).

### Effect of rabbit anti-vimentin polyclonal antibodies on *E. tenella* sporozoite invasion of DF-1 cells

**Fig. 5:**
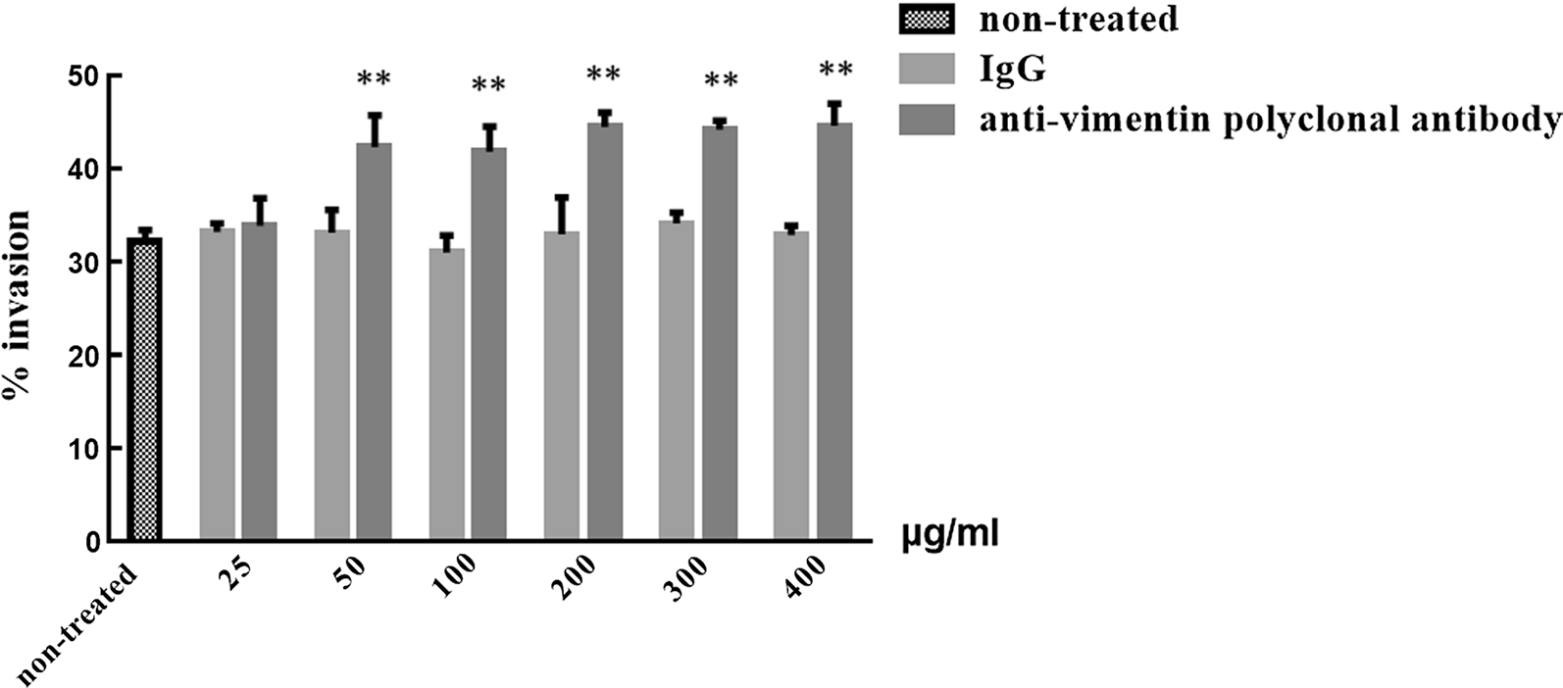
Effect of anti-vimentin polyclonal antibody on *E. tenella* sporozoite invasion in DF-1 cells. **P < 0.01 by Student’s *t*-test compared with anti-vimentin polyclonal antibody treatment or with the same dose of normal rabbit IgG

The sporozoite invasion rate increased significantly after treatment with rabbit anti-vimentin polyclonal antibodies compared with the same dose of normal rabbit IgG-treated and untreated DF-1 cells (*F*_(6,14)_ = 62.015, *P* < 0.0001). As compared with the same dose of naive rabbit sera IgG (used as negative controls), pretreatment at concentrations of 50–400 μg/mL significantly affected the invasion capacity of sporozoites. However, increasing the rabbit anti-vimentin polyclonal antibody concentration produced no significant change in the sporozoite invasion rate (*F*_(4,10)_ = 2.050, *P* = 0.163), indicating that 50 μg/ml concentration of antibody could achieve a good promotion effect (Fig. 5).

### Effects of downregulating vimentin expression on *E. tenella* sporozoite invasion of DF-1 cells

**Fig. 6:**
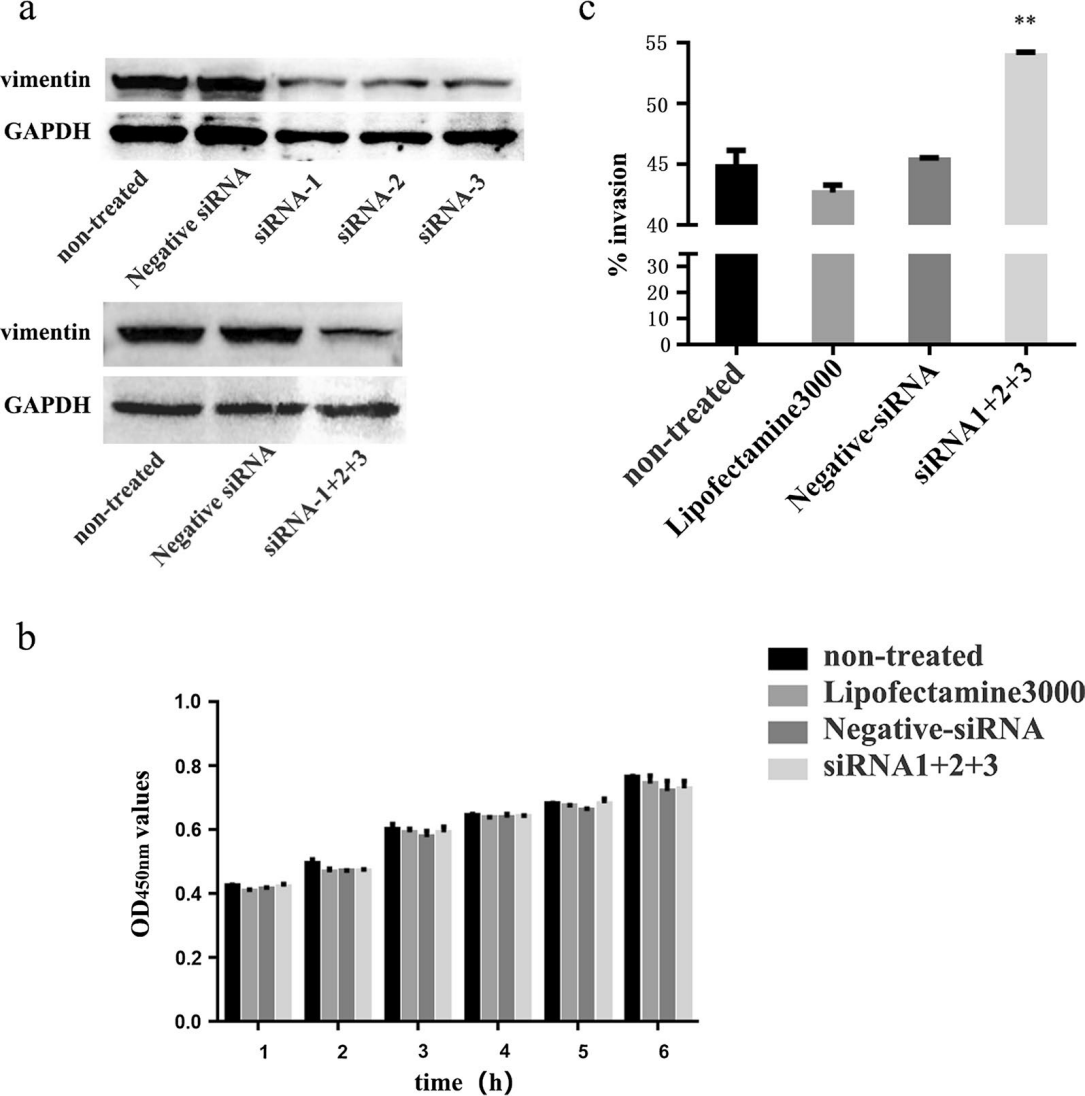
Effects of vimentin downregulation on sporozoite invasion in DF-1 cells. **a** The expression of vimentin protein in different transfection groups. **b** Cell growth curves of different transfection groups. **c** The invasion rate of sporozoites after downregulation of vimentin. **P < 0.01 by Student’s *t*-test compared with downregulated vimentin expression level or the untreated group

Western blot analysis of the interference effect of three siRNAs found that siRNA-1, siRNA-2, and siRNA-3 produced better interference effects than the untreated and negative siRNA control groups, and significantly downregulated vimentin protein expression 24 h after transfection (Fig. 6a). To determine the effect of downregulated vimentin expression level on cell activity and sporozoite invasion rate, DF-1 cells were transfected with siRNA-1, siRNA-2, and siRNA-3. The results showed that compared with the untreated group, treatment with siRNA-1 + 2 + 3, Lipofectamine 3000, or negative siRNA had no significant effect on cell activity (Fig. 6b) (*P* > 0.05), but the invasion rate of sporozoites in the siRNA-1 + 2 + 3-treated group increased significantly (*F*_(1,4)_ = 174.781, *P* < 0.0001), and the increase in Lipofectamine 3000-treated and negative siRNA-treated groups was not significant (Fig. 6c) (*F*_(2,6)_ = 1.494, *P* = 0.298).

## Discussion

Vimentin is a type III intermediate filament protein that is highly evolutionarily conserved in vertebrates and has no homologous proteins in coccidia. Vimentin, together with microtubules and microfilaments, constructs the network structure of protein fibers in cells, which is essential for the maintenance of cell morphology, intracellular material transport, and cell migration [21]. Besides these complex biological functions, vimentin is expressed on the cell surface and acts as a receptor to interact with the proteins of pathogens, regulating the pathogen infection process in host cells. For instance, some viruses and bacteria can bind to vimentin on the cell surface through specific virulence proteins or adhesion factors to facilitate their invasion of host cells [22, 23].

We used qPCR and western blotting to detect the mRNA transcriptional and protein expression levels of vimentin after *E. tenella* sporozoite invasion. The results showed that the transcription and expression levels of vimentin increased significantly in DF-1 cells 6–72 h after infection with sporozoites. Studies have shown that reticuloendotheliosis virus (REV) infection leads to upregulation of vimentin expression in chick embryo fibroblasts (CEF) [20], and *Mycobacterium tuberculosis* infection causes the downregulation of vimentin expression levels in macrophages [24]. In CoS 7 cells infected with *Toxoplasma gondii*, the expression levels of vimentin were dynamic, first showing a decreasing trend, then an increase and subsequent decrease, and finally an increase [14]. The changes in vimentin expression levels differed among different pathogen infections, which suggests that vimentin may play different roles in different pathogen infections.

We used indirect immunofluorescence to detect the distribution of vimentin in the process of sporozoite infection. The results showed that after 24 h of infection, the vimentin accumulated around the sporozoites in DF-1 cells, which was similar to results found in other pathogens. Vimentin rearrangements occurred around parasitophorous vacuole membrane (PVM) after 1 h of *T. gondii* invasion of host cells [25]. Early in African swine fever virus (ASF) and hantavirus infection, vimentin showed stellate aggregation on one side of the Vero cells and then rearranged into a cage structure around the virus replicator [26, 27]. Based on our results, combined with those of existing studies, we speculate that this aggregation may be a protective mechanism for host cells against the spread of pathogens in the cytoplasm. However, the role of vimentin rearrangement in pathogen infection is unclear.

We found that the total phosphorylation level of vimentin changed dynamically in DF-1 cells within 72 h of sporozoite infection. Our previous phosphorylation proteomics results also confirmed that phosphorylation levels at multiple sites in vimentin changed after sporozoite infection. Previous studies have found that the infection process of many Apicomplexa phylum parasites was accompanied by the rearrangement of the host cytoskeleton, and this dynamic change in the cytoskeleton was closely related to the parasite invasion efficiency [7, 28–32]. The change in vimentin distribution is influenced by solubility, which is regulated by phosphorylation [33, 34]. Therefore, we speculated that sporozoites may affect the host cytoskeleton structure by regulating the phosphorylation level of vimentin, creating favorable conditions for invasion and intracellular parasitism.

To further investigate the role of vimentin in DF-1 cells infected with sporozoites, we used an invasion assay in vitro to observe the effect of vimentin polyclonal antibodies or downregulating the expression of vimentin in DF-1 cells on sporozoite invasion. We found that both treatments significantly promoted sporozoite invasion, indicating that vimentin may inhibit *E. tenella* sporozoite invasion. Our results were consistent with those from *T. gondii*, but different from those for bacteria and viruses. Studies have shown that vimentin antibodies inhibited the *Listeria monocytogenes* invasion of human cerebral microvascular endothelial cells (hCMEC) and the survival of *M. tuberculosis* in macrophages [24, 35]. The knockout of vimentin can promote the invasion of *T. gondii* in hCMEC but has no significant effect on intracellular proliferation and release [14]. Silencing or destroying the vimentin protein structure can inhibit the replication and proliferation of avian reticuloendothelial proliferative virus in CEF cells [20], while for dengue virus type 2, vimentin knockout can promote the invasion of hCMEC [36]. These results suggest that vimentin plays different roles in the process of infection of different pathogens. After invasion, *E. tenella* sporozoites round up into trophozoites at about 36 h post-infection and undergo schizogony (asexual multiple fission) at 48–72 h post-infection, resulting in the production of multiple first-generation merozoites. Both mRNA transcriptional and protein expression levels of vimentin increased significantly during these periods, but its specific role in the intracellular development of *E. tenella* sporozoites needs further study.

To sum up, *E. tenella* sporozoite invasion caused changes in host vimentin distribution, and vimentin inhibited the sporozoite invasion of DF-1 cells. However, the specific role of vimentin in coccidian infection remains to be further investigated.

## Supplementary Information

**Additional file 1: Figure S1.** Expression, purification, and validation of vimentin recombinant protein. **a** Lane M: protein marker; Lane 1: negative control (not induced with IPTG); Lanes 2: induced with IPTG for 2 h. **b** Lane 1: purified vimentin detected by SDS-PAGE. **c** Lane 1: purified vimentin verified by western blotting.

## Data Availability

Not applicable.

## Abbreviations

NCBI: National Center for Biotechnology Information
BLAST: Basic Local Alignment Search Tool
MOI: Multiplicity of infection
