# Supplementary figures and images for: Effects of host vimentin on Eimeria tenella sporozoite invasion
Source: Parasit Vectors. 2022 Jan 4;15:8. doi: 10.1186/s13071-021-05107-4 (PMC8729122; doi:10.1186/s13071-021-05107-4)

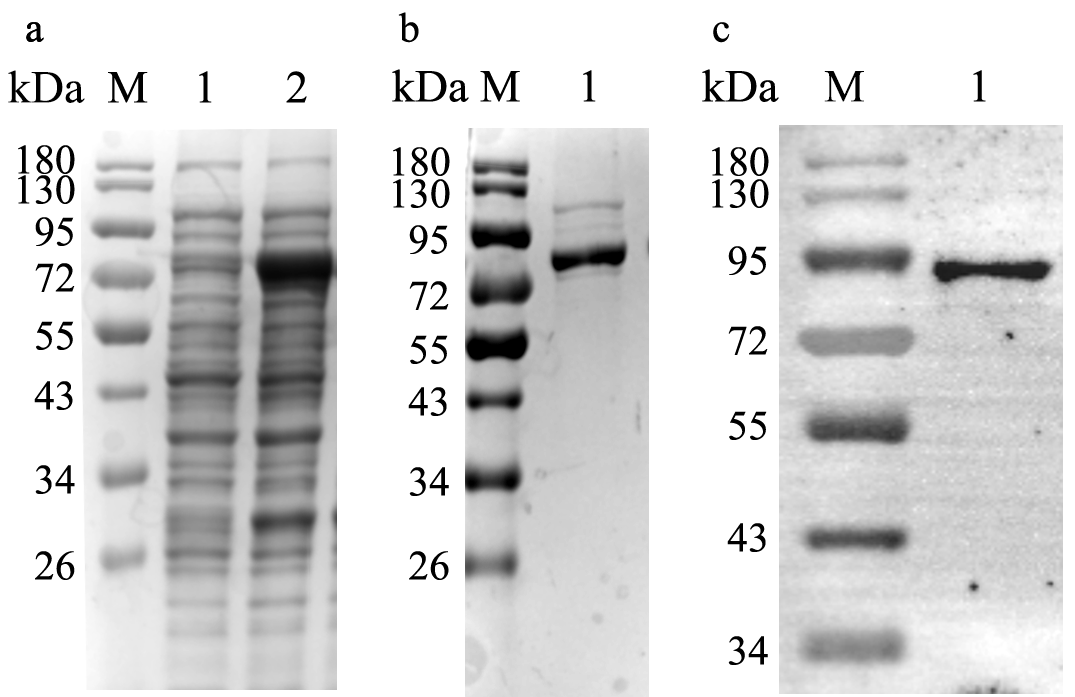

Supplement: Supplementary file 1 — Additional file 1: Figure S1. Expression, purification, and validation of vimentin recombinant protein. a Lane M: protein marker; Lane 1: negative control (not induced with IPTG); Lanes 2: induced with IPTG for 2 h. b Lane 1: purified vimentin detected by SDS-PAGE. c Lane 1: purified vimentin verified by western blotting. [file 13071_2021_5107_MOESM1_ESM.tif]
